# Fast Track Adaptation of Oncolytic Coxsackie B3 Virus to Resistant Colorectal Cancer Cells - a Method to Personalize Virotherapy

**DOI:** 10.1186/s12575-024-00237-2

**Published:** 2024-04-25

**Authors:** Leslie Elsner, Lisanne Heimann, Anja Geisler, Babette Dieringer, Klaus-Peter Knoch, Luisa Hinze, Karin Klingel, Michel Solimena, Jens Kurreck, Henry Fechner

**Affiliations:** 1 Department of Applied Biochemistry, Institute of Biotechnology, Technische Universität Berlin, Straße des 17. Juni 135, 10623 Berlin, Germany; 2 Paul Langerhans Institute Dresden and German Center for Diabetes Research (DZD e.V.), Helmholtz Munich at University Hospital and Faculty of Medicine, Technische Universität Dresden, Dresden, Germany; 3 Cardiopathology, Institute for Pathology and Neuropathology, University Hospital Tuebingen, Liebermeisterstr. 8, 72076 Tübingen, Germany

**Keywords:** Cancer Therapy, Oncolytic Virus, Personalized Therapy, Colorectal Carcinoma, Direct Virus Evolution, Coxsackievirus B3

## Abstract

**Background:**

The efficacy of oncolytic viruses (OV) in cancer treatment depends on their ability to successfully infect and destroy tumor cells. However, patients’ tumors vary, and in the case of individual insensitivity to an OV, therapeutic efficacy is limited. Here, we present a protocol for rapid generation of tumor cell-specific adapted oncolytic coxsackievirus B3 (CVB3) with enhanced oncolytic potential and a satisfactory safety profile. This is achieved by combining directed viral evolution (DVE) with genetic modification of the viral genome and the use of a microRNA-dependent regulatory tool.

**Methods:**

The oncolytic CVB3 variant PD-H was adapted to the refractory colorectal carcinoma cell line Colo320 through serial passaging. XTT assays and virus plaque assays were used to determine virus cytotoxicity and virus replication in vitro. Recombinant PD-H variants were generated through virus mutagenesis. Apoptosis was detected by Western blots, Caspase 3/7 assays, and DAPI staining. The therapeutic efficacy and safety of the adapted recombinant OV PD-SK-375TS were assessed in vivo using a subcutaneous Colo320 xenograft mouse model.

**Results:**

PD-H was adapted to the colorectal cancer cell line Colo320 within 10 passages. Sequencing of passage 10 virus P-10 revealed a heterogenous virus population with five nucleotide mutations resulting in amino acid substitutions. The genotypically homogeneous OV PD-SK was generated by inserting the five detected mutations of P-10 into the genome of PD-H. PD-SK showed significantly stronger replication and cytotoxicity than PD-H in Colo320 cells, but not in other colorectal carcinoma cell lines. Increase of apoptosis induction was detected as key mechanisms of Colo320 cell-specific adaptation of PD-SK. For in vivo safety PD-SK was engineered with target sites of the miR-375 (miR-375TS) to exclude virus replication in normal tissues. PD-SK-375TS, unlike the PD-H-375TS not adapted homolog suppressed the growth of subcutaneous Colo320 tumors in nude mice without causing any side effects.

**Conclusion:**

Taken together, here we present an optimized protocol for the rapid generation of tumor cell-specific adapted oncolytic CVB3 based on the oncolytic CVB3 strain PD-H. The protocol is promising for the generation of personalized OV for tumor therapy and has the potential to be applied to other OV.

## Background

Oncolytic viruses (OVs) are an innovative class of tumor-selective immunotherapeutics that selectively replicate in tumor cells. The anti-tumor efficiency is achieved through the virus induced destruction of the tumor cells leading to a potent systemic anti-tumor immune response [1]. Among the numerous OVs tested in clinical trials, three have received clinical approval: Oncorine [2], an engineered adenovirus which is used in combination with chemotherapy for the treatment of nasopharyngeal and head and neck cancers, T-Vec [3], an engineered herpes simplex virus (HSV) that overexpresses granulocyte-macrophage colony-stimulating factor for the treatment of advanced melanoma, and Delytact [4], a triple-mutated, replication-conditional HSV for the treatment of malignant glioma. However, in regard to the therapeutic efficacy of all clinical trials involving OVs from 2000 to 2020, only about 20% of treated cancer patients derived benefit from OV treatment [5]. Although the exact reasons have not yet been elucidated in detail, both viral and host-dependent factors appear to be responsible for the failure of OV therapy in cancer patients. OVs developed in recent decades typically exhibit a broad tumor cell tropism [6]. This trait arises from the inherent tropism of the viruses to tumor cells, but also from modification implemented in the viruses by genetic engineering [7]. Nevertheless, multiple studies with hundreds of tumor cell lines and various in vivo tumor models have shown that the sensitivity of tumor cells to OVs varies widely, ranging from highly sensitive to completely resistant [8–12]. The latter concerns tumor cells from tumors of different entities, but also those belonging to tumors of the same entity [10, 12, 13]. The heterogeneity of tumor cells can directly impact the therapeutic success of OV therapy. This is due to inefficient viral delivery and viral entry into the cancer cells as well as the limited viral replication and release and the virus-induced destruction of the tumor cells. Consequently, this impairs the induction of a systemic antitumor immune response, which is a key mechanism determining the oncolytic efficacy of OV [1].

Directed virus evolution (DVE) is an approach employed to enhance the efficacy of OV. Although DVE has been widely used by virologists to study the life cycle and evolution of viruses [14, 15] it is still rarely used for development of improved OV. DVE bases on the ability of viruses to rapidly generate mutations in their genetic material while replicating. This enables viruses to adapt to host cells that do not or only partially support virus replication through an autonomous process [16]. Compared to genetically engineering, a major advantage of DVE is that improved OV can be generated without the need to elucidate the underlying resistance mechanisms of the cancer cells [17]. Key features of such adapted OV are increased oncolytic activity and viral fitness in the refractory cancer cells [10]. However, depending on the goal of the adaptation strategy, adapted OVs may also exhibit higher stability, better intratumoral spread, or a wider range of infectivity than the parental viruses [10, 18, 19]. Both DNA and RNA viruses has been used to develop novel OVs by DVE [10, 20, 21]. Although it has been shown that an adapted OV generated by DVE can also has a higher oncolytic activity in other tumor cell lines than in the one in which it was adapted [10], the adaptation process is generally tumor cell-specific, and the OV has typically the highest oncolytic activity in the tumor cells in which it was generated [22, 23]. Therefore, DVE is a technology that could be particularly suitable for the development of OV in the context of personalized cancer therapy. One major obstacle to the widespread use of this technology in OV production is the inability to predict whether the customized OV will retain the safety features of its parent strain. Additionally, the process of developing and testing the customized OV is complex, time-consuming and expensive [21].

Here, we have developed a protocol for the rapid generation of tumor cell-specific adapted OV with high safety using the OV PD-H, a non-enveloped single (+)-strand RNA virus of the picornavirus family which belongs to the coxsackie B virus of the serotype 3 (CVB3) [24]. Key points of this protocol are the strict restriction of the number of passages in the resistant tumor cell line to 10, direct cloning of detected mutations within the viral genome into the cDNA of PD-H and equipping of the genome of the adapted virus with target sites of miR-375 (miR-375TS), to prevent possible virus induced side effects in vivo. We show that using this protocol PD-H can be easily and fast adapted to the refractory colorectal cancer cell line Colo320. The adapted virus PD-SK replicated up to 100-fold stronger in Colo320 cells and induced significantly stronger cells lysis than the parental founder in vitro. In vivo, PD-SK engineered with miR-375TS demonstrated distinct oncolytic activity in Colo320 tumors without inducing side effects, whereas the parental founder remains ineffective. Mechanistically, the stronger activity of the adapted virus was primarily due to the increased induction of apoptosis in Colo320 target cells.

## Materials and Methods

### Cell Culture

HeLa cells were cultured in Eagle’s minimal essential medium (MEM) (Gibco, Karlsruhe, Germany) supplemented with 5% fetal calf serum (FCS), 0,02 M HEPES, 1% non-essential amino acids (NEAA) and 1% penicillin / streptomycin (P/S). HEK293T cells were cultured in DMEM High glucose (Biowest, Darmstadt, Germany) supplemented with 10% FCS, 1% P/S, 1% L-glutamine and 1% Na-pyruvate. The murine colorectal carcinoma cell lines Colon-26 and the human colorectal cancer cell lines DLD-1, Colo320, Colo205 and Colo680H were cultured in RPMI 1640 (c.c.pro, Oberdorla, Germany) supplemented with 10% FCS, 1% P/S, 1% L-glutamine and 2% Na-pyruvate. The human colorectal cancer cell line Caco-2 cells was cultured in DMEM High glucose (Biowest) supplemented with 10% FCS, 1% P/S, 1% L-glutamine, 1% NEAA and 2% Na-pyruvate. The murine colorectal CT-26Luc cells were cultured in RPMI 1640 (c.c.pro) supplemented with 10% FCS, 1% P/S, 1% L-glutamine, 1% NEAA and 2% Na-pyruvate.

### Cell Killing Assay

1.5 × 10^5^ Colo320 cells and 5 × 10^4^ HeLa cells were seeded in 96-well plates and infected the next day with virus at a dose of MOI 0.1, 1 and 10 in 100 µl. After 1 h the medium was removed and 100 µl fresh medium was added. 24 h and 48 h later the medium was removed, cells were fixed with 10% trichloroacetic acid (TCA) (Carl Roth, Karlsruhe, Germany) and stained with 30 µl crystal violet solution (Carl Roth). The cells were washed three times with phosphate buffered saline (PBS) and then photographed.

### Serial Passaging of PD-H in Colo320 Cells

Colo320 cells were seeded in 24-well (passage 1–8, each 8 × 10^5^ cells), 12-well (passage 9, 1.6 × 10^6^ cells) or 6-well (passage 10, 4 × 10^6^ cells) plates. After 24 h, the medium was removed, and cells were infected with PD-H at MOI 0.1 in fresh medium for 1 h. Cells were washed with PBS and fresh culture medium was added (24-well 500 µl, 12-well 1 ml, 6-well 2 ml). After incubation for 72 h viruses were isolated by three freeze-thaw cycles, cell debris was removed by centrifugation and virus titer in supernatant was determined by plaque assay on HeLa cells. The isolated virus batches were named according to their passage. The viruses were stored at -80 °C until use in the next round of passaging. In each further round 0.1 MOI of virus obtained from the previous round of passaging was applied to fresh Colo320 cells. In total 10 virus batches (PD-1 to PD-10) were generated.

### Virus Plaque Assay

HeLa cells were seeded in 24-well plates as confluent monolayers. After 24 h, medium was removed, and cells were incubated for 30 min with 300 µl of serial 10-fold dilutions of virus-containing samples in PBS. After removing the supernatant cells were overlaid with 3.2% BD-Difco Noble Agar (Thermo Fisher Scientific, Waltham, USA) containing Eagle’s minimal essential medium (MEM) (Gibco). Seventy-two h after virus infection HeLa cells were stained with Tetrazoliumbromid-Iodnitrotetrazoliumchlorid solution (both VWR International GmbH, Darmstadt, Germany) and incubated for 4 h before plaque counting.

### Virus Growth Curves

For generation of viral growth curves, 1.5 × 10^5^ Colo320 cells were seeded in 96-well plates. After 24 h, medium was removed, and cells were infected with 100 µl virus suspension at MOI 0.1 and incubated for 1 h. Afterwards the medium was removed and 200 µl fresh medium was added. Cells were disrupted by three freeze-thaw cycles 0, 4, 8, 24, 48 and 72 h post infection. The cell lysate was centrifuged, and the supernatant was used for determination of virus titers by plaque assay on HeLa cells.

### Cell Viability Assay

Cell viability was assessed by using Cell Proliferation Kit (XTT) (Promega GmbH, Walldorf, Germany) according to the manufacturer’s instructions. Cells were seeded in 96-well plates and infected the next day when they reached a confluence of about 80%. Twenty-four h, 48–72 h after infection absorbance levels were measured using the TriStar2 LB 942 Modular Multimode Microplate Reader (Berthold Technologies, Bad Wildbad, Germany). As negative control, cells were treated with 50 µl 5% Triton X-100 solution.

### Mutagenesis and Cloning of PD-H cDNA-containing Plasmids

Mutagenesis was done by the In-Fusion HD Cloning Kit (Takara Bio, Shiga, Japan) according to manufacturer’s instructions. The mutagenesis-primers were designed using the online infusion primer designing tool (Takara Bio). Eight different plasmids were generated by mutagenesis of the plasmid pJet-CVB3-PD-H [24] containing the cDNA of the oncolytic CVB3 PD-H [24]. These plasmids were named as follows and contain nucleotide substitutions leading to substitution of certain amino acids (aa) in the viral polyprotein (shown in parenthesis), pJET-CVB3-PD-K4 (A3T), pJET-CVB3-PD-K1 (E596V, E768D), pJET-CVB3-PD-K2A (Y940C), pJET-CVB3-PD-K2B (N1027D), pJET-CVB3-PD-K2AB (Y940C, N1027D), pJET-CVB3-PD-K1-2AB (E596V, E768D, Y940C, N1027D), pJET-CVB3-PD-SK (A3T, E596V, E768D, Y940C, N1027D). A further plasmid, pJet-CVB3-PD-SK-375TS (A3T, E596V, E768D, Y940C, N1027D) was generated by mutagenesis of the plasmid pJet-CVB3-PD-H-375TS [24] containing the PD-H cDNA with two target sites of the miR-375 in the 3’ UTR [24].

### Generation of Recombinant Viruses

HEK293T cells were seeded into 6-well plates and next day at a confluence of 70–80% transfected with 2.5 µg of plasmids containing viral cDNA using PEImax (Polysciences Europe GmbH, Hirschberg an der Bergstraße, Germany). The viruses PD-H and PD-H-375TS were generated by transfection of the plasmids pJET-CVB3-PD-H [24] and pJET-CVB3-PD-H-375TS [24]. The viruses PD-K1, PD-K4, PD-K2A, PD-K2B, PD-K2AB, PD-K1-2AB, PD-SK, and PD-SK-375TS were generated by transfection of plasmids containing the corresponding viral cDNAs. Seventy-two h after transfection cells were disrupted by three freeze-thaw-cycles. The cell lysate was centrifuged, and the supernatant was used for determination of virus titers by plaque assay on HeLa cells. Viruses were amplified by infection of HEK293T with 0.3 MOI of viruses. For in vivo studies PD-H-375TS and PD-SK-375TS were purified and concentrated by ultracentrifugation with 30% sucrose gradient as described previously [25].

### Sequencing of CVB3 Genome

The whole genome of PD-5 and PD-10, and all mutated sites of the engineered PD-H variants were sequenced. Therefore, viral RNA was extracted with the NucleoSpin RNA Virus Kit (Macherey-Nagel, Düren, Germany) according to the manufacturer’s instructions and reverse transcribed by using the High-Capacity cDNA Reverse Transcription Kit (Applied Biosystems, Foster City, USA). PCR fragments were generated with Q5® High-Fidelity DNA Polymerase (New England Biolabs, Frankfurt, Germany) using CVB3 specific primers and sequenced by sanger sequencing by LGC Biosearch Technolgies (Berlin, Germany). Sequence alignment was done with SnapGene 5.1.7 software.

### Comparison of aa Substitutions Found in PD-10 with Other CVB3 Strains

PD-H according to [24]. The other strains can be found at the National Center for Biotechnology Information (NCBI) with the following accession numbers. Strain 0 AY752945.1; strain 20 M88483.1; strain 2035 A KY286529.1; strain 28 AY752944.2; strain 31-1-93 AF231763.1; strain H3 U57056.1; strain L M16572.1; strain M2 M33854.1; strain Nancy JX312064.1; strain P AF231764.1 and strain RD HQ157560.1.

### Virus Attachment and Uptake Assay

Colo320 cells were seeded in 24-well plates. Next day when cells reached a confluence of 80% the cells were washes with ice cold PBS, then incubated with 0.1 MOI virus in a volume of 500 µl ice cold cell culture medium on ice for 1 h. The medium was removed, the cells washed twice with ice cold PBS. To determine virus attachment thereafter the cells were immediately frozen to -80 °C. After three freeze-thaw-cycles the supernatant was used for viral RNA extraction. To determine virus uptake the cells were infected as described, overlaid with 500 µl cell culture medium and incubated for further 30 min at 37° C. The medium was removed, the cells were washed twice with icecold PBS and total RNA from cells was isolated using TRIZOL reagent (Life Technologies, Carlsbad, USA). RT-PCR was used to determine virus genome copy number.

### Determination of Virus Genome Copy Number by Quantitative RT-PCR

For quantification of viral genomic RNA, total RNA from virus infected cells was isolated using the NucleoSpin RNA Virus Kit (Macherey-Nagel) or using TRIZOL reagent (Life Technologies) according to the manufacturer’s instructions and reverse transcribed by using the High-Capacity cDNA Reverse Transcription Kit (Applied Biosystems). Real-time PCR was performed using the CVB3 specific forward primer, 5’-CCCTGAATGCGGCTAATCC and the reverse primer 5’- ATTGTCACCATAAGCAGCCA in SsoFastTM EvaGreen Supermix (Bio-Rad Laboratories, Hercules, USA). Cycle times were one cycle at 50 °C for 2 min followed by 94 °C for 10 min and 40 cycles at 94 °C for 15s and 60 °C for 60s. A standard curve was used to calculate the number of viral genome copies. The RNA copies were determined by the ΔΔ_Ct_ calculation method.

### Western Blots

Colo320 cells were seeded in 6-well plates. Twenty-four h later when cells reached a confluence of 80% the cells were infected with PD-H and PD-SK at MOI 0.1. Twenty-four h later cells were washed with PBS and trypsinated. Cell suspension was centrifuged, supernatant removed, and cell pellet immediately frozen at -80 °C. Cells were treated with lysis buffer (20 mM TRIS/HCl, pH 8.0, 140 mM NaCl, 1 mM EDTA, 1% Triton X-100, 1% protease inhibitor cocktail) (Sigma-Aldrich, Taufkirchen, Germany) and 1% phosphatase inhibitor cocktail (Calbiochem, San Diego, USA). Protein concentration was measured by a BCA assay (Thermo Fisher Scientific). Cell extracts were separated by SDS/PAGE and immunoblotted with primary antibody anti-γ-tubulin (T6557) (Sigma-Aldrich), selfmade mAb against CVB5-VP1 which binds CV-1 of CVB3 [26], anti-eIF4G (N-20) (Santa Cruz Biotechnology, Dallas, Texas, USA), anti-cleaved caspase 3 (Asp175, #9661), anti-caspase 8 (1C12, #9746), anti-caspase 9 (C9, #9508) and anti-PARB (#9542) (all Cell Signaling Technology, Danvers, MA, USA). The monoclonal anti-VP1 antibody was generated against VP1 from CVB5 strain Faulkner. For detection the membrane was blocked with 5% dry milk/PBS-T and incubated overnight at 4 °C with the respective antibodies. Subsequently the membrane was washed three times with PBS-T and incubated with goat anti-mouse and anti-rabbit IgGs conjugated to horseradish peroxidase (Bio-Rad, Hercules, USA) in 5% dry milk/PBS-T for 1 h. Magic MarkXP (Thermo Fisher Scientific) was used as a molecular weight marker to determine size of detected proteins after western blotting. Chemiluminescence was performed using the Supersignal West Pico Substrate (Thermo Fisher Scientific) and detected with Imager 600 from GE Healthcare (Chalfont St Giles, UK). Quantification of the expression of indicated genes was carried out relative to the expression of γ-tubulin by densitometric analysis using the ImageJ densitometry software.

### Fluorescence Imaging

Colo320 cells were seeded in 24-well plates and were infected the next day when cells reached a confluence of about 80% with PD-H and PD-SK at MOI 0.1. Twenty-four h after infection cells were fixed with 4% formaldehyde (Carl Roth) for 15 min, washed three times with PBS and incubated with 500 µl blocking solution (PBS, 5% goat serum, 0.3% Triton X-100) for 1 h. Blocking solution was removed, and cells were incubated with primary antibody for cleaved caspase-3 (Cell Signaling Technology) at 4 °C overnight. After washing with PBS, incubation with secondary antibody Goat anti-Rabbit IgG (H + L) Alexa Fluor™ 488 (Thermo Fisher Scientific) was carried out for 2 h. Thereafter, cells were washed three times with PBS followed by co-staining with 1 µg/mL of 40,6-diamidino-2-phenylindole (DAPI, Sigma-Aldrich) for 10 min. Cells were washed twice again with PBS and images were taken by fluorescence microscopy (Observer Z1, Carl Zeiss, Oberkochen Germany).

### Caspase-3/7 Activities

Caspase-3/7 activities were measured using Caspase-Glo® 3/7 Assay Systems (Promega GmbH) according to the manufacturer’s instructions. Cells were seeded in 96-well plates and were infected the next day when cells reached a confluence of about 80% at MOI of 0.1 and 0.01. Twenty-four h after infection luminescence was measured using the TriStar2 LB 942 Modular Multimode Microplate Reader (Berthold Technologies, Bad Wildbad, Germany).

### Quantification of MicroRNA Levels

For quantification of miR-375 level, total RNA from cells or mouse tissues was isolated by using TRIZOL reagent (Life Technologies). RNA was reverse transcribed by using the High-Capacity cDNA Reverse Transcription Kit (Applied Biosystems). Expression levels of miR-375 (assay ID: 000564) were determined by utilizing the TaqMan gene expression master mix and specific TaqMan gene expression assays (Life Technologies) according to the manufacturer’s instructions. The data were analyzed by using the ΔΔCt method, and results were normalized against U6 snRNA (assay ID: 001973) levels of cell lines and tissues.

### MicroRNA-dependent Virus Silencing

HEK293T cells were seeded in 24-well plates and transfected the next day when they reached a confluence of about 70–80% with 800 ng of the plasmid pCMV-miR-375 expressing the miR-375 or the plasmid pCMV-GFP-miR-216 expressing GFP protein and the miR-216. Both plasmids were purchased from Origene Technologies (Rockville, MD, USA). Transfection was done using PEImax (Polysciences Europe GmbH). Forty-eight h post transfection cells were inoculated with PD-SK-375TS at MOI 0.01 for 30 min at 37 °C. After removal of viral solutions, fresh medium was added. Twenty-four h post infection cells were disrupted by three freeze-thaw-cycles, the cell lysate was centrifuged, and the virus containing supernatant was used for determination of virus titers by plaque assay in HeLa cells.

### Xenografted Subcutaneous Colo320 Cancer Mouse Model

The animal experiments were performed in accordance with the principles of laboratory animal care and all German laws regarding animal protection and approved by the responsible local authorities (State Office of Health and Social Affairs, Berlin, Germany, reference number G 0048/18). For generation of Colo320 tumors, 6-week-old female Balb/C nude mice (Charles River, Sulzfeld, Germany) were injected with 5 × 10^6^ cells subcutaneously into the right and left flank. After 9 days, when tumor reached a diameter of approximately 5 mm, one of the tumors was injected with 3 × 10^6^ PFU of PD-H-375TS or PD-SK-375TS, each in 30 µl PBS. Control animals were injected with 30 µl PBS. Tumor volume was measured every two days. The maximal tumor size/burden permitted by ethics committee was 1,8 cm^3^. The maximal tumor size/burden was not exceeded in the experiments.

### Histopathological Analysis

For histopathological analysis tissues were fixed with 4% paraformaldehyde (Carl Roth) and embedded in paraffin. The tissue was cut in 5 μm-thick sections and stained with hematoxylin and eosin (H&E) to visualize inflammation and tissue destruction.

### Statistical Analysis

Statistical analysis was performed with Graph-Pad Prism 8.2 Software. Results are expressed as the mean SEM for each group. Statistical significance was determined by use of the two-tailed unpaired Student’s t-test for cell culture investigations and by use of the Mann-Whitney U-test for in vivo investigations. Differences were considered significant at *p* < 0.05.

## Results

### Generation of PD-SK by Direct Evolution of PD-H in Colo320 Cells and Engineering of the PD-H cDNA Clone

**Fig. 1:**
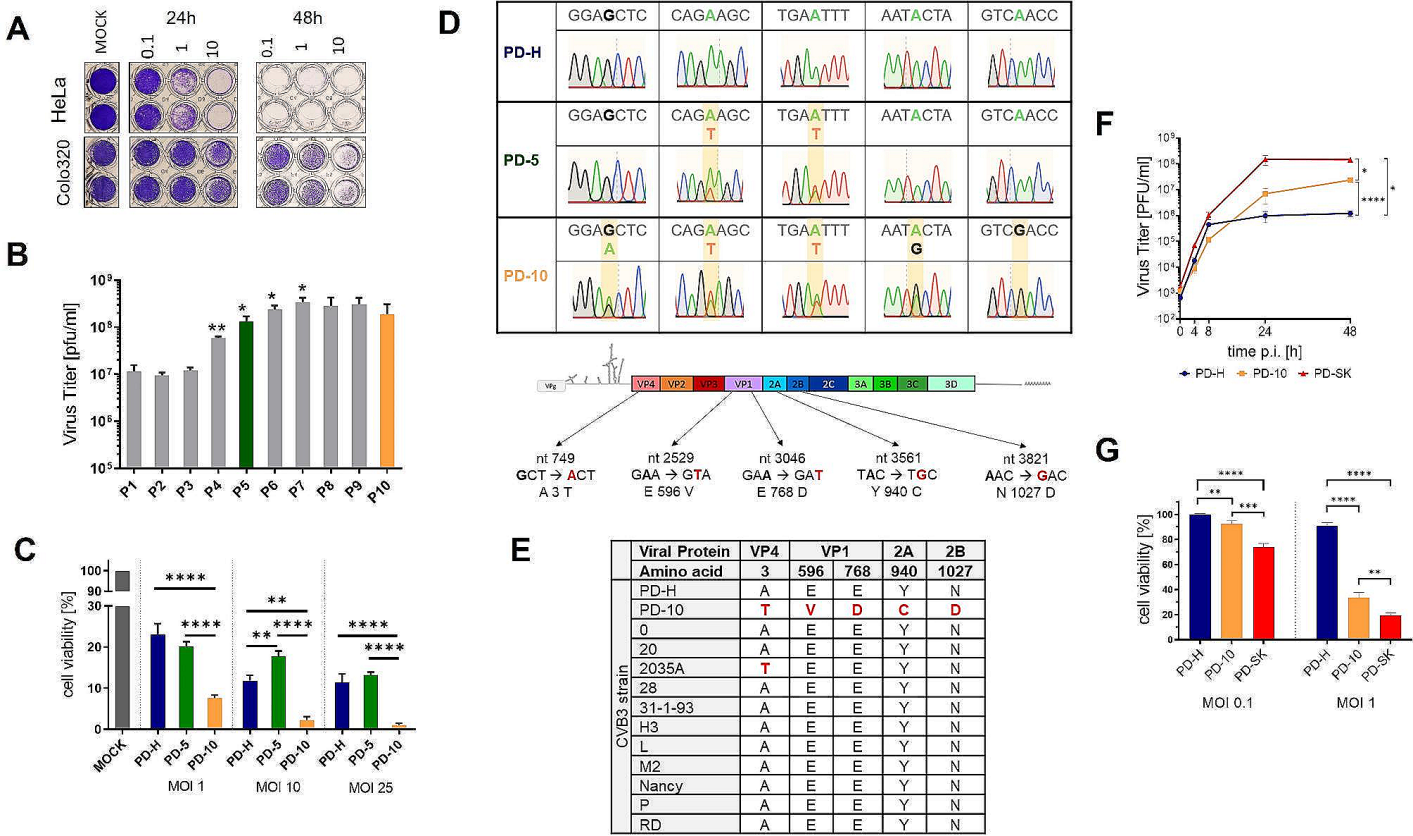
Adaption of PD-H in Colo320 and functional analysis of PD-SK. (**A**) Susceptibility of Colo320 cells to PD-H. Colo320 and HeLa cells were infected as indicated with of PD-H. Cell viability was determined 24 h and 48 h post infection by crystal violet staining. (**B**) Development of viral titers during serial passaging of PD-H. Colo320 cells were infected with MOI 0.1 and incubated for 72 h. Viral titer was determined by plaque assay. The procedure was repeated for 10 passages. Shown are mean values ± SEM. Significance compared to previous passage, * *p* < 0.05; ** *p* < 0.01. (**C**) Changes of cell viability during passaging of PD-H. Colo320 cells were infected with PD-H, PD-5 or PD-10 at the indicated MOIs. Cell viability was measured by XTT assay 72 h after infection. Shown are mean values ± SEM. Significance, ** *p* < 0.01; **** *p* < 0.0001. (**D**) Detection of mutations in PD-5 and PD-10. *Upper panel*, Chromatograms of the sanger sequencing of viral RNA isolated from PD-H, PD-5 and PD-10. Nucleotide substitutions are highlighted in yellow boxes. *Lower panel*, schematic of PD-H genomic RNA, location of the nucleotide and resulted aa substitutions detected in PD-10. (**E**) Comparison of aa substitutions found in PD-10 with other CVB3 strains. (**F**) Virus growth kinetics. Colo320 cells were infected with PD-H, PD-10 and PD-SK at MOI 0.1. Virus titer was determined by plaque assay. Shown are mean values ± SEM. Significance compared to PD-H, * *p* < 0.05; **** *p* < 0.0001. (**G**) Cell viability. Colo320 cells were infected with PD-H, PD-10 or PD-SK at the indicated MOI. Cell viability was measured 48 h after infection. Shown are mean values ± SEM. Significance, ** *p* < 0.01; *** *p* < 0.001; **** *p* < 0.0001

The oncolytic CVB3 variant PD-H [24], which is in the focus of this study, was generated from a cDNA clone isolated from the oncolytic CVB3 variant PD-0, which demonstrated potent oncolytic activity in colorectal carcinomas as described by our group [13]. However, we have also observed that certain colorectal cancer cell lines, including Colo320 cells, exhibit resistance to PD-0 [13]. To confirm that Colo320 cells are also refractory to PD-H and therefore a suitable target for the DVE approach, we infected Colo320 cells and the control cell line HeLa with the virus and analyzed the cell viability 24 and 48 h later. HeLa cells were highly sensitive to PD-H, while Colo320 cells were lysed only at a high viral dose (Fig. 1A), confirming the insensitivity of Colo320 cells to PD-H. To adapt PD-H to Colo320 cells, we serially passaged PD-H in Colo320 cells using a low MOI of 0.1. Passage 1 virus (PD-1) was harvested, and virus titers determined by plaque assay. Fresh Colo320 cells were than infected with 0.1 MOI of PD-1 and ten virus passages were performed. A significant increase in virus titers was detected beginning at passage 4 up to passage 7, where the maximum was achieved (Fig. 1B). To assess the cytotoxic activity of passaged viruses, XTT cell viability assay was performed for viruses harvested at passage 5 (PD-5) and passage 10 (PD-10) and compared them to PD-H. The cytotoxicity of PD-5 was similar to PD-H, while the cytotoxicity of PD-10 was much stronger than that of PD-H and PD-5 indicating that the increase in cytotoxic activity occurred with a time delay during adaptation of PD-H to Colo320 cells (Fig. 1C).

To determine the genetic basis of virus adaptation, the viral genome of PD-5 and PD-10 was sequenced. For PD-5, two mutations, both in the capsid protein VP1 were detected leading to aa substitutions at aa26 [E → V] and aa198 [E → D]). In PD-10 besides aa26 and aa198 three additional aa substitutions were found, in the viral capsid protein VP4 (aa3 [A → T]), the viral protease 2 A (aa89 [Y → C]) and the viroporin 2B (aa26 [N → D]) (Fig. 1D). All mutations were unusual for CVB3, as there was nearly no homology to other CVB3 isolates (Fig. 1E). Importantly, sanger sequencing detected signals of mutated and founder sequences at the site of nucleotide substitution for both mutation sites of PD-5 and in four of the five mutation sites of PD-10 (Fig. 1D) demonstrating that both PD-5 and PD-10 represent a heterogenous virus population. We hypothesized that all aa substitutions were beneficial for the virus to replicate and kill Colo320 cells. Therefore, we directly cloned the five mutations detected in PD-10 into cDNA of PD-H using the rapid in Fusion snap assembly cloning procedure. The resulted viral cDNA was transfected into HEK293T cells, and the virus PD-SK was produced. To determine whether PD-SK retained the functional features of PD-10, we compared its growth kinetics by generating one step growth curves and measured its cytotoxicity by XTT assays in Colo320 cells. As expected, PD-SK replicated to distinctly higher virus titers and induced distinctly higher cytotoxicity compared to those of PD-H, but surprisingly virus titers and cytotoxicity of PD-SK were also significantly higher than those of PD-10 (Fig. 1G, F), indicating that the virus clone was more potent than PD-10.

### Increase of Oncolytic Efficiency of PD-SK is Restricted to Colo320 Cells

**Fig. 2:**
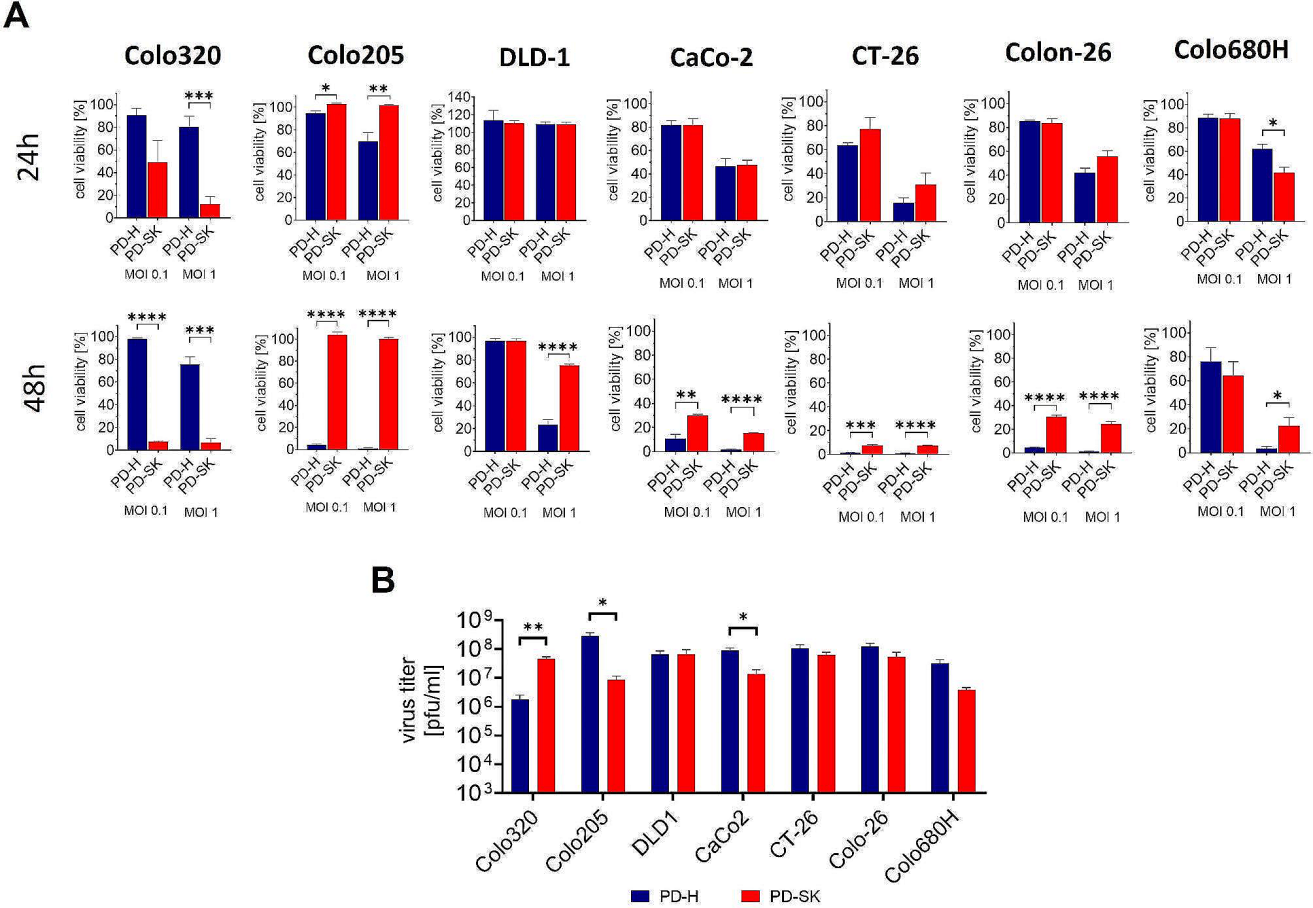
Comparison of oncolytic efficiency and replication of PD-H and PD-SK in colorectal cancer cell lines. (**A**) Cell viability. The colorectal cancer cell lines Colo320, Colo205, DLD-1, CaCo-2, CT-26, Colon-26 and Colo680H were infected with PD-H and PD-SK at indicated MOIs; cell viability was measured 24 h and 48 h after infection. Shown are mean values ± SEM. Significance, * *p* < 0.05, ** *p* < 0.01; *** *p* < 0.001; **** *p* < 0.0001. (**B**) Virus titer. Cells were infected with MOI 1; virus titer was determined 48 h after infection by plaque assay. Shown are mean values ± SEM; Significance, * *p* < 0.05; ** *p* < 0.01

DVE leads to target cell-specific virus adaptation. However, it has been observed that the adaptation of an OV to one tumor cell line can also improve its oncolytic efficiency in other tumor cells [10, 19]. To find out whether this also applies to PD-SK, we determined the cytotoxicity of PD-SK and PD-H in six additional colorectal cancer cell lines 24 h and 48 h after infection with 1 and 0.1 MOI and investigated the replication of both viruses in these cell lines 24 h after infection with 1 MOI. In five of the six cell lines, the cytotoxicity of PD-SK was moderately lower than that of PD-H, while the sixth cell line, Colo205, which was also sensitive to PD-H, could not be lysed by PD-SK (Fig. 2A). In four of six colorectal cancer cell lines PD-SK replication was similar or minimally reduced compared to PD-H, whereas in two cell lines, Colo205 and CaCo2, the replication of PD-SK was distinctly lower than those of PD-H (Fig. 2B). These results show that the increased oncolytic activity of PD-SK in Colo320 did not lead to increased oncolytic efficiency in other colorectal carcinoma cell lines. On the contrary, DVE of PD-H in Colo320 cells had a negative effect on replication and cytotoxicity in other cancer cell lines.

### Acquired Virus Mutations act Cooperatively to Achieve Best Performance of the Adapted Virus

**Fig. 3:**
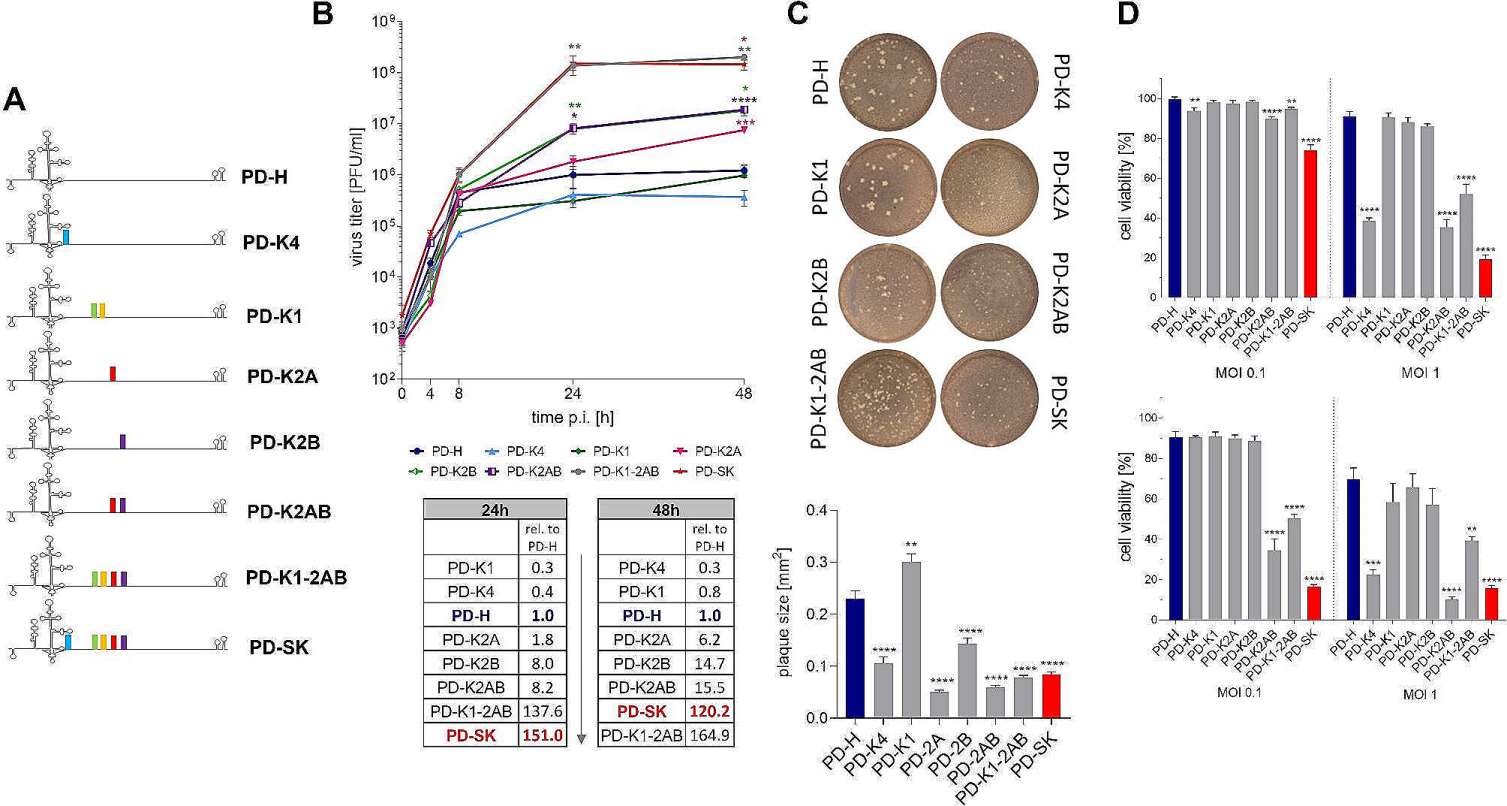
Importance of individual aa substitutions for the replication and cytotoxicity PD-SK. (**A**) Schematic representation of generated PD-H mutant variants. The following mutants were generated. PD-K4 (A3T), PD-K1 (E596V, E768D), PD-K2A (Y940C), PD-K2B (N1027D), PD-K2AB (Y940C, N1027D), PD-K1-2AB (E596V, E768D, Y940C, N1027D) and PD-SK (A3T, E596V, E768D, Y940C, N1027D), the parenthesis shows the respective aa exchanges compared to PD-H. Colored columns in the scheme represent the location of nucleotide substitutions compare to PD-H. (**B**) Virus growth kinetics. *Upper panel*, Colo320 cells were infected with PD-H, PD-K4, PD-K1, PD-K2A, PD-K2B, PD-K2AB, PD-K1-2AB and PD-SK at MOI 0.1. Virus titers were determined by plaque assay at indicated time points. Shown are mean values ± SEM. Significance compared to PD-H, * *p* < 0.05; ** *p* < 0.01; *** *p* < 0.001; **** *p* < 0.0001. *Lower table*, replication of the virus variants after 24 h and 48 h relative to PD-H. (**C**) Plaque sizes. Plaque sizes of indicated viruses were determined by plaque assays on HeLa cell monolayers. *Upper panel –* shows representative images of viral plaques (white dots) *Lower panel -* Graphical representation of plaque diameter. Shown are mean values ± SEM. Significance compared to PD-H, ** *p* < 0.01; **** *p* < 0.0001, for *n* = 50 plaques. (**D**) Cell viability. Colo320 cells were infected with indicated viruses at MOI 0.1 and 1; cell viability was measured 24 h (*upper diagram*) and 48 h (*lower diagram*) after infection. Shown are mean values ± SEM. Significance compared to PD-H, ** *p* < 0.01; *** *p* < 0.001; **** *p* < 0.0001

To determine the importance of acquired mutations for the improved performance of PD-SK in Colo320 cells, we next generated a panel of viral mutants with single or clustered mutations in the PD-H background (Fig. 3A) and compared their replication and cytotoxicity with PD-H and PD-SK. The virus mutant PD-K1-2AB containing the mutated VP1, 2A and 2B proteins, exhibited the best growth among the tested virus mutants. It replicated similarly to PD-SK with approximately 150-fold higher final virus titers compared to PD-H. The virus variants PD-K2B and PD-K2AB, which contain the mutated 2B and the mutated 2A and the mutated 2B, respectively, had virus titers about 15 times higher than PD-H, but did not reach the replication of PD-SK, while the variant PD-K2A replicated only slightly better than PD-H. In contrast, the viruses PD-K4 and PD-K1, containing the mutated VP4 and the mutated VP1, respectively underperformed and replicated distinctly worse than PD-H (Fig. 3B). With the exception of PD-K1, whose plaques were larger than those of PD-H, all mutant viruses and PD-SK had smaller plaques than PD-H (Fig. 3C). The cytotoxicity of the virus mutants was highly variable and only partially correlated with their replication in Colo320 cells. Only PD-K4, PD-K2AB and PD-K1-K2AB demonstrated greater cytotoxicity than PD-H but did not reach the cytotoxicity observed for PD-SK. Meanwhile PD-K1, PD-K2A and PD-K2B were as ineffective as PD-H in killing Colo320 cells (Fig. 3D). In summary, these data demonstrate that each mutation acquired during evolution of PD-H in Colo320 cells affected virus replication and cytotoxicity. When considered individually, the effects were either positive or negative. Interestingly, mutations that on their own did not improve or even impaired the oncolytic activity of the virus, in combination with other mutations, contributed to a further increase in the oncolytic activity of the adapted virus.

### Enhancement of Apoptosis Induction is a Crucial Mechanism Determining the Improved Oncolytic Efficiency of PD-SK in Colo320 Cells

**Fig. 4:**
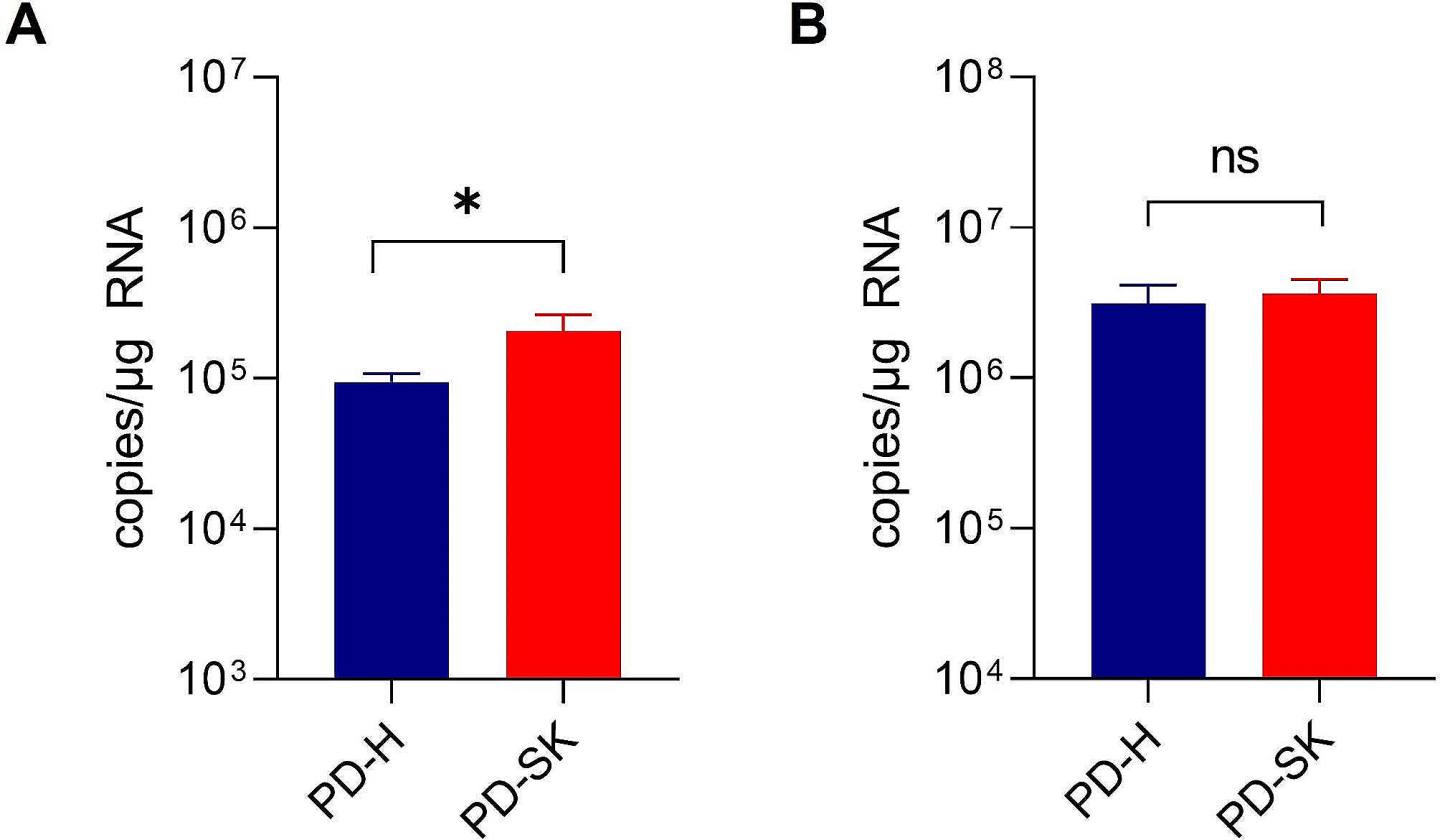
Attachment and uptake of PD-SK and PD-H to Colo320 cells. Colo320 cells were and infected with 0.1 MOI of PD-SK and PD-H. (**A**) Virus attachment. After infection the cells on ice, virus was removed, and cells were washed two times with ice-cold PBS and viral RNA isolated. Virus genome copy number was determined by quantitative RT-PCR. Shown are mean values ± SEM, Significance, * *p* < 0.05. (**B**) Virus uptake. After infection the cells on ice with PD-H and PD-SK Colo320 cells were incubated at 37 °C for 1 h. Cells were washed two times with ice-cold PBS and virus RNA isolated. Virus genome copy number was determined by quantitative RT-PCR. Shown are mean values ± SEM. Significance, * *p* < 0.05; ** *p* < 0.01

Next, we were interested in determining the mechanisms involved in overcoming resistance of Colo320 cells by PD-SK. Mutations acquired during evolution of PD-H in Colo320 cells affected the viral capsid proteins VP1 and VP4 and the non-structural proteins 2 A and 2B. Both capsid proteins are involved in mediating the virus entry into target cells [27], suggesting that mutation within these proteins may affectPD-SK entry into Colo320 cells. To prove this Colo320 cells were treated with 0.1 MOI PD-SK and PD-H and the virus attachment and internalization were investigated. PD-SK bound significantly better to the surface than PD-H, although the overall effect was small. However, this did not resulted in increased uptake of PD-SK into the cells (Fig. 4A, B). suggesting that viral entry does not play a critical role in the final adaptation of PD-SK.

**Fig. 5:**
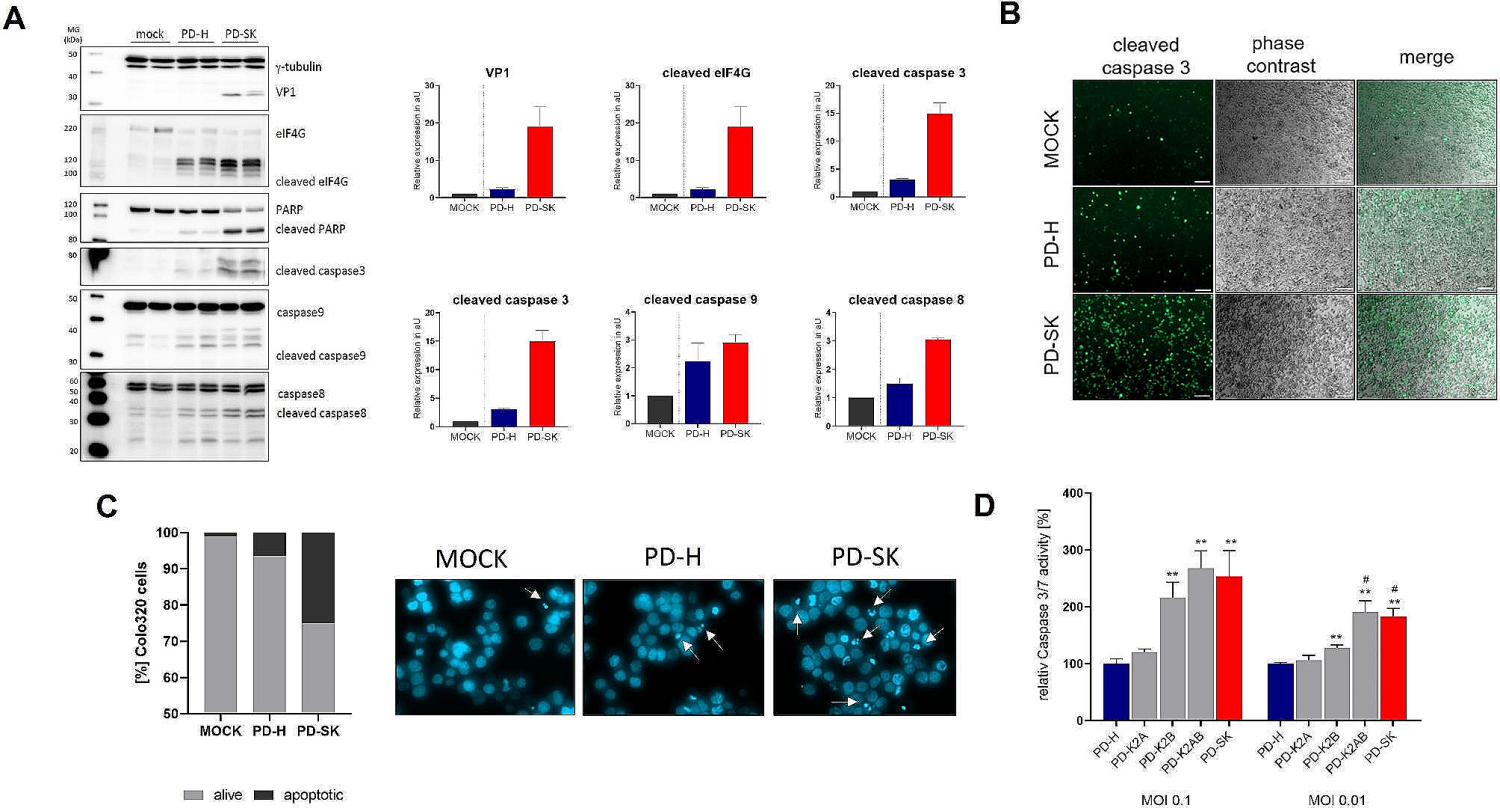
PD-SK induces stronger apoptosis in Colo320 cells than PD-H. (**A**) Determination of CVB3 VP1 and cellular proteins involved in apoptosis induction. *Left images*, Colo320 cells were infected with 0.1 MOI of PD-SK or PD-H and analyzed 24 h later for expression of eIF4G, cleaved eIF4G, PARP, cleaved PARP and cleaved Caspase 3, Caspase 9, cleaved caspase 9, caspase 8 and cleaved caspase 8 by western blotting. The γ-tubulin was used as internal loading control. *Right diagrams*: Quantification of the expression of indicated proteins was carried out relative to the expression of γ-tubulin by densitometric analysis using the ImageJ densitometry software. (**B**) Immunofluorescence staining of Colo320 cells for cleaved caspase 3. Cells were seeded in 24 well plates and infected with PD-H and PD-SK at MOI 0.1. Twenty-four h after infection cells where fixed with 4% formaldehyde and stained for cleaved caspase 3. Scale bar 100 μm. (**C**) Dapi staining of Colo320 cells. Cells were treated as in B and stained with Dapi. *Left diagram*, relative amount of living and apoptotic cells of 100 random counted cells. *Right*, images after staining with Dapi. Arrows indicate apoptotic cells. Scale bar 20 μm. (**D**) Relative caspase 3/7 activity in Colo320 cells 24 h after infection with PD-H, PD-K2A, PD-K2B, PD-K2AB and PD-SK at indicated MOIs. Shown are mean values ± SEM. Significance compared to PD-H, * *p* < 0.05; ** *p* < 0.01. Significance compared to PD-K2B: # *p* < 0.05

The CVB3 proteins 2A and 2B have distinct functions in the life cycle of coxsackieviruses, but a common feature is their involvement in regulation of apoptosis [28, 29]. Accordingly, we investigated apoptosis induction in Colo320 cells 24 h after infection with 0.1 MOI PD-H and PD-SK. As shown by western blotting, increased expression of cleaved eukaryotic initiation factor 4 g (elF4G), cleaved poly ADP-ribose polymerase (PARP) and cleaved caspase-3, -8, and 9 was detected in PD-SK-infected compared to PD-H-infected cells (Fig. 5A). The stronger induction of apoptosis by PD-SK compared to PD-H was confirmed by detection of higher expression of cleaved caspase 3 by immunostaining (Fig. 5B) as well as by increase of chromatin condensation and nuclear fragmentation by DAPi staining (Fig. 5C). To determine whether increase of apoptosis was caused by the action of mutated 2 A or mutated 2B protein, next we infected Colo320 cells with 0.1 MOI PD-K2A, PD-K2B, PD-K2AB, PD-SK and PD-H, respectively, and measured the caspase 3/7 activity 24 h later. Compared to PD-H, no change in caspase 3/7 activity was detected in PD-K2A-infected cells, whereas a significant increase was observed after infection with PD-K2B. An even higher caspase 3/7 activity, reaching values measured for PD-SK, was detected in PD-K2AB-infected Colo320 cells (Fig. 5D), indicating that the cooperative action of mutated 2 A and 2B enhanced apoptosis in Colo320 cells. Thus, the enhanced apoptosis induction seems to be key mechanisms determining the higher oncolytic activity of PD-SK in Colo320 cells.

### Genetic Engineering of PD-SK with miR-375TS Inhibits Virus Replication in miR-375 Expressing Target Cells

**Fig. 6:**
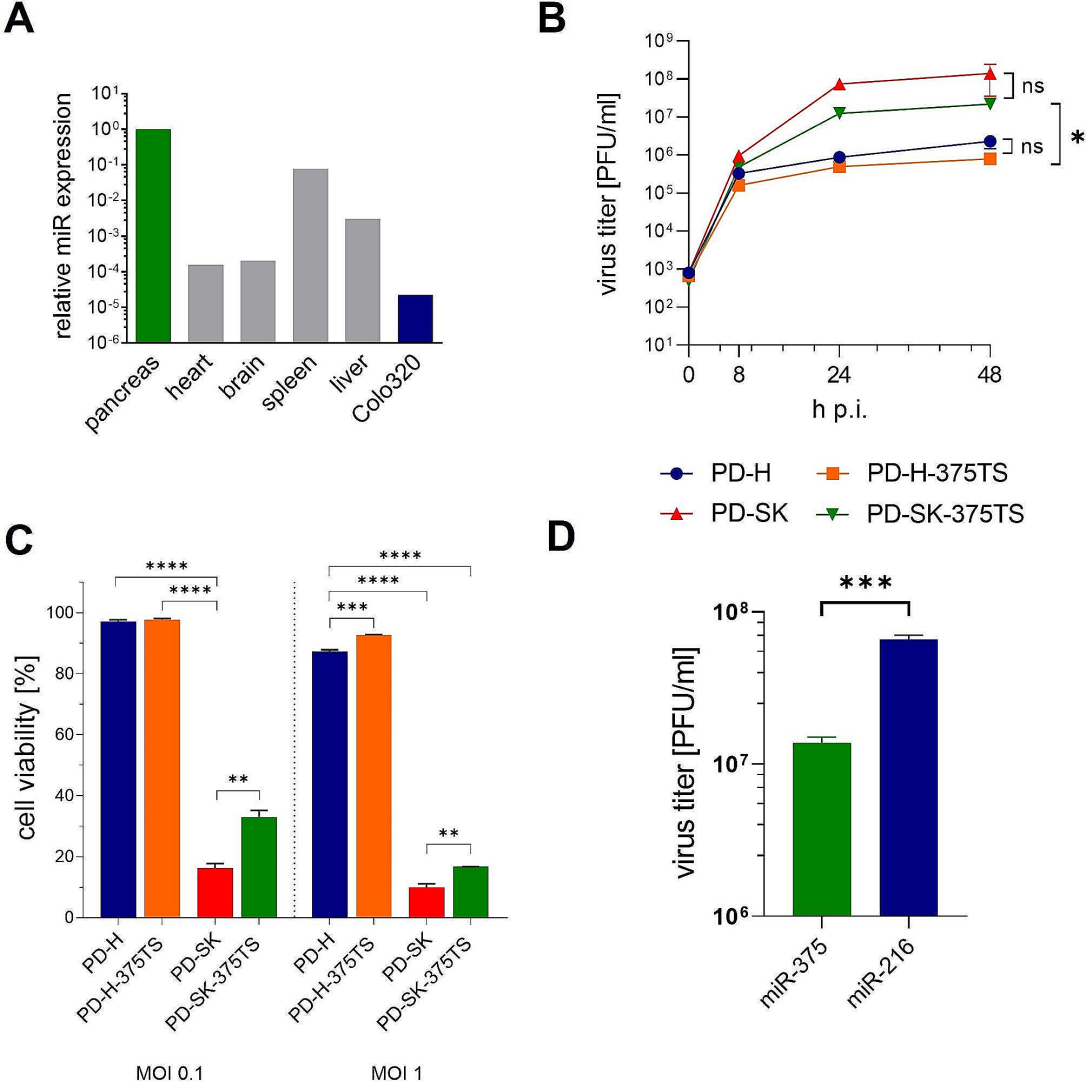
Replication, cytotoxicity of PD-SK-375TS in Colo320 cells and its sensitivity to miR-375. (**A**) Relative expression level of miR-375 in Colo320 cells and murine organs. Expression levels were determined by quantitative RT-PCR. Each miR expression level was normalized against the level of endogenous U6 snRNA expression and is shown relative to miR-375 levels of the pancreas which was set to 1. (**B**) Virus growth kinetics. Colo320 cells were infected with PD-H, PD-H-375TS, PD-SK or PD-SK-375TS at MOI 0.1. Virus titer was measured by plaque assay at indicated time points. Shown are mean values ± SEM. Significance, * *p* < 0.05; n.s., not significant. (**C**) Cell viability. Colo320 cells were infected with PD-H, PD-H-375TS, PD-SK or PD-SK-375 at the indicated MOI. Cell viability was determined by XTT assay 48 h after infection. Shown are mean values ± SEM. Significance, ** *p* < 0.01; *** *p* < 0.001; **** *p* < 0.0001. (**D**) Silencing of PD-SK-375TS by miR-375. HEK293T cells were transfected with pCMV-miR-216a or pCMV-miR-375 and infected 48 h later with MOI 0.01 PD-SK-375TS. Virus Virus titers were determined by plaque assay 24 h post infection. Shown are mean values ± SEM. Significance, *** *p* < 0.001

Although the evolution of PD-H led to the adaptation of the virus to Colo320 cells, it cannot be ruled out that the adapted PD-SK virus could also replicate in normal body cells and thus cause side effects after application in vivo. To ensure the safety of the virus, we have therefore generated PD-SK-375TS containing miR-375TS the 3’ UTR of its genome. MiR-375 is a pancreas-specific microRNA that inhibits the replication of oncolytic CVB3 equipped with miR-375TS in the normal organs of mice [30]. However, to ensure that the virus continues to replicate in Colo320, a prerequisite for the success of this approach is that miR-375 is not expressed in Colo320 cells, or only in small quantities. This could be confirmed by measurement of miR-375 expression by RT-PCR in Colo320 cells using real time RT-PCR (Fig. 6A). To assess the functionality of PD-SK-375TS, we next examined its cytotoxicity and replication in Colo320 cells and compared it with PD-SK, PD-H as well as a further control virus PD-H-375TS, representing PD-H equipped with miR-375TS. PD-SK-375TS showed slightly lower replication and cytotoxicity than PD-SK, but replicated and killed Colo320 cells significantly stronger than PD-H or PD-H-375TS (Fig. 6B, C). In addition, PD-SK-375TS replication was distinctly inhibited in HEK293T cells transfected with miR-375, indicating that the virus became sensitive to miR-375 (Fig. 6D).

### PD-SK-375TS Reduces Growth of Subcutaneous Colo320 Tumors in Xenografted Mice Without Inducing Side Effects

**Fig. 7:**
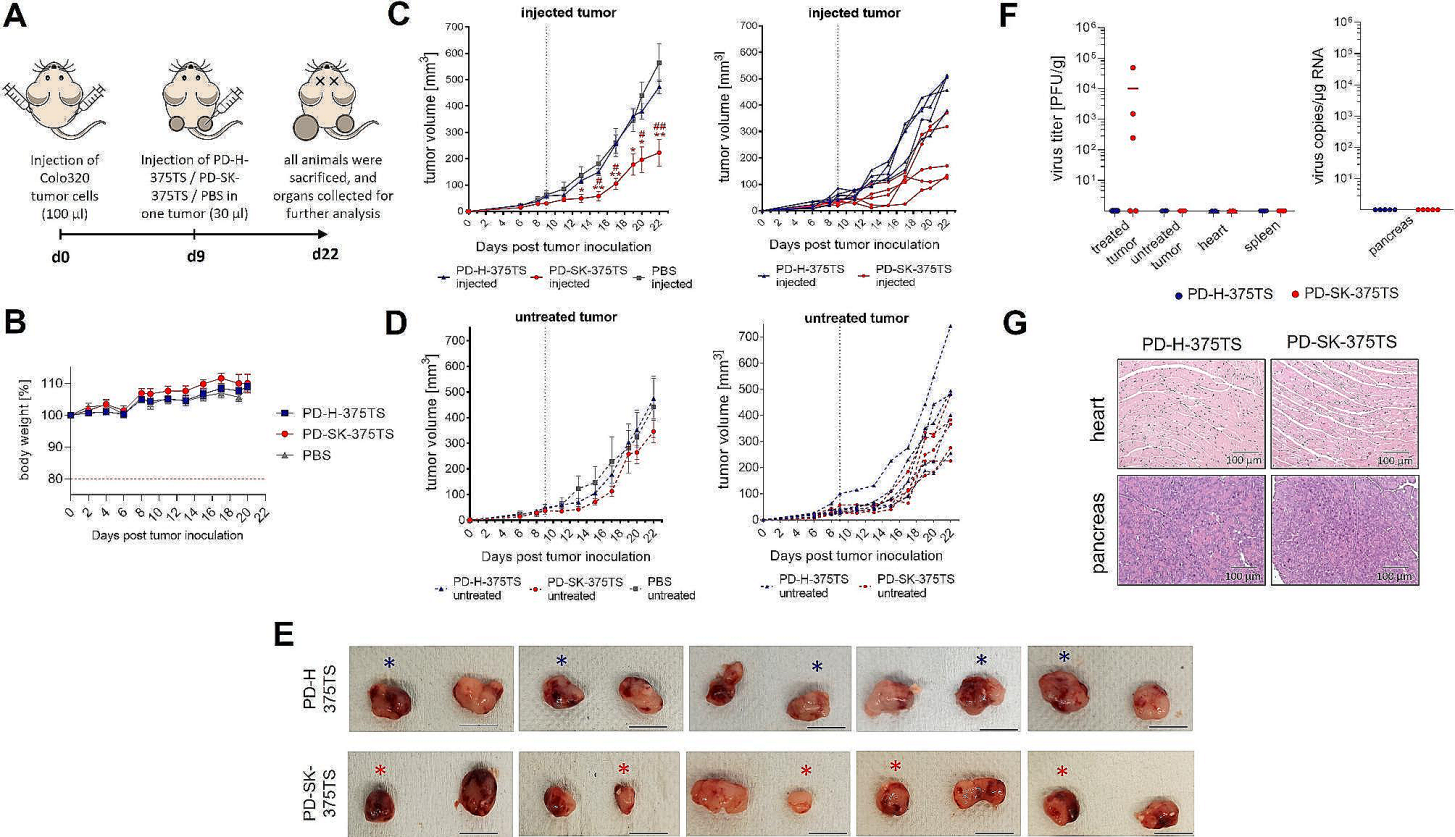
Oncolytic activity and safety of PD-SK-375TS and PD-H-375TS in nude mice with Colo320 cell tumors. Colo320 tumors were established on both flanks of Balb/C nude mice, when the tumor size reached ∼ 0.5 cm, one of the tumors was injected with 3 × 10^6^ PFU PD-SK-375TS (*n* = 5) or PD-H-375TS (*n* = 5) or with PBS (*n* = 5) while the contralateral tumor remained untreated. All animals were sacrificed 13 days later and investigated. (**A**) Time course of virus application and analysis. (**B**) Development of animal weight. (**C**) Growth of the injected tumor. *Left*, shows mean values ± SEM for each group; *right*, data from PD-SK-375TS and PD-H-375TS of the left diagram for each animal. Significance PD-SK-375TS vs. PD-H-375TS, * *p* < 0.05; ** *p* < 0.01. Significance, PD-SK-375TS vs. PBS, ^#^*p* < 0.05; ^##^*p* < 0.01. The dashed lines indicate the times at which the viruses were injected. (**D**) Growth of the non-injected contralateral tumor. *Left*, shows mean values ± SEM for each group; *right*, data from PD-SK-375TS and PD-H-375TS of the left diagram for each animal. (**E**) Representative images of explanted left and right tumors of PD-SK-375TS and PD-H-375TS; * marks the injected tumor. Scale bar = 1 cm. (**F**) Distribution of PD-SK-375TS and PD-H-375TS in indicated organs and in the injected and untreated contralateral tumors. *Left diagram*: PD-H-375TS and PD-SK-375TS titers determined by plaque assay on HeLa cells. *Right diagram*: Virus genome copy number in the pancreas determined by quantitative RT-PCR. (**G**) Histological examination of heart and pancreas. Shown are representative tissue slides after staining with H&E

Next, we compared the oncolytic efficiency and safety of PD-SK-375TS in vivo in a subcutaneous xenograft mouse model of Colo320 cancer. Colo320 tumor cells were injected into both flanks of nude mice. When the tumor reached a size of about 0.5 cm, one tumor was injected with 3 × 10^6^ PFU of PD-SK-375TS, the control virus PD-H-375TS or with PBS. The contralateral tumor was not injected (Fig. 7A). During the 22-day study period, a similar increase in body weight was observed in all treated animal groups (Fig. 7B). No deaths or impaired health status were detected in any of the animals. Treatment with PD-SK-375TS resulted in a significant reduction in the growth of the injected Colo320 tumors, while no growth inhibition was noted in the tumors injected with PD-H-375TS (Fig. 7C, E) and in non-injected tumors. (Fig. 7D, E). Thirteen days after virus injection all animals were sacrificed, and the abundance of the viruses was determined in the tumors and organs. Neither PD-SK-375TS nor the PD-H-375TS were detected in the heart and the pancreas of the mice as well as in the spleen, and the untreated tumors. PD-H-375TS was also not detected in the injected tumor. In contrast, in three of five treated animals PD-SK-375TS was found in the injected tumor (Fig. 7F). In accordance with the lack of virus replication, no pathological alterations were detected in the pancreas and the heart, representing the most susceptible organs for CVB3, for both viruses (Fig. 7G). These data demonstrate, that Colo320 tumors became susceptible to PD-SK-375TS in vivo. Moreover, PD-SK-375TS did not induce side effects in the treated animals.

## Discussion

**Fig. 8:**
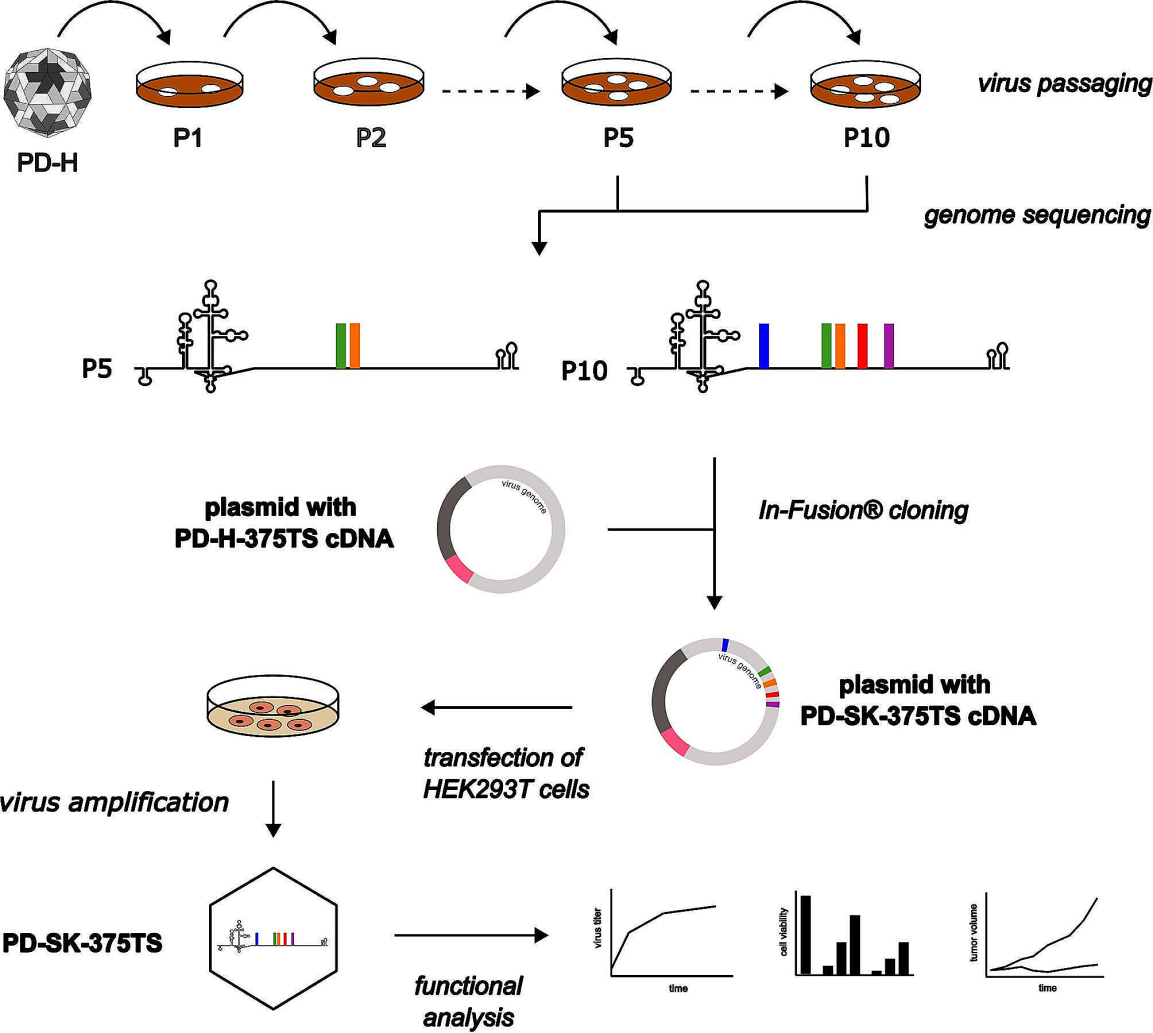
Schematic of the steps required to generate and analyze PD-SK-375TS from PD-H

Here we present a protocol for rapid development of tumor cell specific-adapted oncolytic CVB3 with enhanced oncolytic potential and a satisfactory safety profile (Fig. 8). The protocol consists of a standardized method of DVE, combined with genetic engineering of the viral genome of the founder virus and use of a microRNA-dependent regulation tool. Starting from the oncolytic CVB3 strain PD-H and the colorectal tumor cell line Colo320 refractory to this OV, we generated PD-SK-375TS using this method. Compared to its founder, the virus showed significantly stronger tumor cell-specific replication and cytotoxicity in Colo320 *in vitro.* Additionally, it efficiently suppressed subcutaneous Colo320 tumor growth in nude mice in vivo without inducing side effects in the treated mice.

Oncolytic virotherapy is a new immunotherapy for cancer. However, clinical trials have shown that only a minority of patients profit from OV treatment. Since efficient infection, replication and damage of tumor cells is crucial for triggering a strong and sustained anti-tumor immune response and thus for the therapeutic outcome, the primary resistance of tumor cells to the virus may be a reason for the failure of this approach. Therefore, the development of improved OVs that are able to overcome the resistance of tumor cells is an indispensable prerequisite for further increasing the efficacy of virotherapy. The mechanisms that lead to viral resistance in tumor cells are often complex and poorly understood, which is why the improvement of OVs by single targeted genetic modification of the viral genome alone is only possible to a limited extent. Additionally, the genetic heterogenicity of tumors require individual solutions for patients. DVE is a method that utilizes the intrinsic activity of viruses to autonomously adapt to a cellular environment that does not primarily support viral infection. This makes DVE an excellent option for the development of OVs for personalized applications. However, one of the biggest challenges in using DVE is to produce an OV with sufficient safety within a defined time period to ensure that the patient can actually benefit from its use. The protocol we established here addresses this question. To restrict time a key point of the developed protocol concerns the limitation of the number of virus passages used to a necessary minimum, which is particularly important in terms of reducing the time required to generate the adapted virus by DVE. Depending on the virus and cell line used as well as the aim of the investigations, the number of passages in the various studies typically varies between 5 and more than 30 [10, 11, 22, 23, 31–33]. In this study, we found that 10 passages were sufficient to adapt the founder virus PD-H to the refractory cell line Colo320 confirmed by the observation that at this passage number the increase of viral replication reached a plateau, and the cytotoxicity of the virus was strongly enhanced. However, sequencing revealed that PD-10 virus population at this time point was heterogenous, demonstrating that the adapted virus had not yet fully prevailed itself. Typically, this observation would require further passaging [23] or in vitro selection of the adapted variant for example by plaque purification to generate a homogenous virus isolate [11, 34]. We demonstrate that these elaborate procedures can be avoided by directly inserting all detected mutations into the genome of the founder virus PD-H using fast and easy to perform site directed mutagenesis of the viral cDNA. Interestingly, the engineered virus PD-SK did not only show improved oncolytic activity compared to the founder but also to PD-10, indicating that engineered PD-SK was better adapted to Colo320 cells than PD-10. Furthermore, the method used to develop PD-SK fixes the adapted virus in a cDNA clone, allowing for repeated virus production using the same DNA starting material.

Mutations acquired within a virus population during an adaptation process can have positive, neutral or negative effects on the function of the virus [35]. In the case of successful evolution in a tumor cell, mutations that negatively affect the virus are not expected to be fixed. Therefore, it was a surprising result, that two of the five detected mutations we detected in PD-10 for itself inhibited virus replication in Colo320 cells and the other three mutations for itself had only minor positive effects on the oncolytic activity of the virus. Only when the five mutations acted in concert in PD-SK viral replication and oncolytic viral activity were fully activated. The latter emphasizes for our protocol the need to insert the entirety of the mutations into the founder virus genome to achieve optimal performance of the engineered OV. This assessment was confirmed by a further experiment we carried out to adapt PD-H to a pancreas carcinoma cell line. Again, the greatest increase in oncolytic activity of the modified adapted virus was achieved when all mutations found in the heterogenous virus population of passage 10 were inserted into the genome of the virus founder (results not shown).

The second important point to consider when creating personalized OVs is the safety of the OVs. Although several studies have shown that adapted OVs do not perform worse than the viral founder in terms of their safety profile [10, 19] it cannot be ruled out a priori that OVs generated by DVE may induce side effects. Therefore, a comprehensive safety analysis is required before these OVs can be used on a patient. However, this would significantly delay the use of the virus or possibly even prevent it if side effects occur. To overcome this drawback, we used an microRNA-dependent technology, that enables cell- and tissue-specific suppression of virus replication [36]. Using this technology, we and others have demonstrated that OVs can efficiently be suppressed in normal tissues, while virus replication and oncolytic activity remains unaffected in targeted tumor cells [24, 30, 36–39]. Importantly, equipping the virus genome with suitable target sites of microRNAs can even convert highly virulent strains of CVB3 avirulent [30]. With this technology, potential side effects of customized OV can be prevented from the outset, rendering extensive safety analyses of DVE-generated viruses unnecessary. In this study, the genome of PD-SK was equipped with miR-375TS, which we found in a previous investigation to be capable of preventing CVB3-induced replication in normal mouse tissues [24, 30]. In fact, we provide evidence here that the virus did not replicate or damage normal tissues in Colo320 tumor-bearing nude mice.

Since the success of DVE depends on various factors, it remains a crucial question whether the protocol developed here can also be used for the adaptation of PD-H to other tumor cells and whether it can be transferred to other OVs. We can answer this question in the affirmative, as we have successfully adapted PD-H to another colorectal and a pancreatic cancer cell line (results not shown). Moreover, a similar protocol was recently successfully utilized to produce an improved oncolytic alphavirus [10], demonstrating that the protocol could be transferred to other OVs as well.

Despite DVE is a mechanism that leads to tumor specific adaptation of viruses, several studies have found that an adapted OV can also become more active in other tumor cells [10, 19], probably as the resistance of the tumor cells to the OV was mediated by similar mechanisms. In this study, the enhanced oncolytic activity of PD-SK was restricted to the Colo320 cell line and was mediated by enhanced virus-mediated induction of apoptosis. Colorectal carcinoma cell lines exhibit high genetic diversity, and among the colorectal cancer cell lines studied here, Colo320 differs significantly from the others in terms of its global expression profile [40]. The latter could explain why PD-SK activity was restricted to Colo320 cells. On the other hand, this result confirms the personalized nature of our approach.

## Conclusion

Tumor cell-specific adapted OVs represent the next generation of OVs with the potential to significantly enhance the efficacy of cancer therapy through personalized application. Previous procedures demonstrating the production of such viruses are often protracted, lack standardization, and necessitate extensive safety testing due to unpredictable changes occurring during the virus adaptation to tumor cells. The standardized protocol we have developed facilitates the rapid development of tumor cell-specific adapted oncolytic CVB3 viruses with enhanced oncolytic activity and a satisfactory safety profile, thus overcoming these challenges. This established protocol can also be applied across different tumor types, transferred to other OVs and allows for further optimization.

## Data Availability

No datasets were generated or analysed during the current study.

## Abbreviations

aa: Amino acid
CVB3: Coxsackie B virus of the serotype 3
DMEM: Dulbecco’s modified Eagel’s medium
DVE: Directed viral evolution
eIF4G: Eukaryotic initiation factor 4 g
FCS: Fetal calf serum
H&E: Hematoxylin and eosin staining
HSV: Herpes simplex virus
MEM: Eagle’s minimal essential medium
miR-375TS: Target sites of microRNA-375
NEAA: Non-Essential Amino Acids
OV: Oncolytic virus
P/S: Penicillin / streptomycin
PARP: Poly ADP-ribose polymerase
PBS: Phosphate buffered saline
TCA: Trichloroacetic acid
TME: Tumor microenvironment
